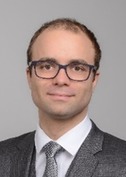# Recent Advances in the Chemistry of Heavier Group 14 Enolates

**DOI:** 10.1002/chem.201902307

**Published:** 2019-09-19

**Authors:** Michael Haas

**Affiliations:** ^1^ Institute of Inorganic Chemistry Technische Universität Graz Stremayrgasse 9/IV 8010 Graz Austria

**Keywords:** germenolates, metal enolates, sila-aldol chemistry, silenolates, stannenolates

## Abstract

Recently heavier Group 14 enolates showed their importance and applicability in a broad range of chemical transformations. They were found to be key intermediates during the synthesis of photoinitiators, as well as during the formation of complex silicon frameworks. This Minireview presents general strategies towards the synthesis of heavier Group 14 enolates (HG 14 enolates). Structural properties, as well as their spectroscopic behavior are outlined. This study may aid future development in this research area.

## Graphical abstract

**Heavy duty**: This minireview presents a summary of synthetic protocols towards the formation of heavier metal enolates. Furthermore, the spectroscopic behavior and the structural data for HG 14 enolates were summarized. In addition, it outlines the recent advances in this research field.

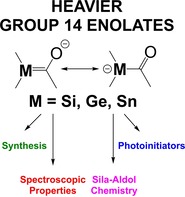

## 1 Introduction

The chemistry of metal enolates is thoroughly investigated and understood to a very high degree.^1^ Moreover, the classical aldol reaction is one of the most important biosynthetic tools for life on earth.^2^, ^3^ Although the first report on the synthesis of heavier Group 14 enolates by Bravo‐Zhivotovskii and co‐workers was in 1989,^4^ the synthesis and characterizations of these derivatives is still a challenging endeavor. In 2003 Ottosson succeeded in isolating a silenolate, which had a high thermal stability, in order to perform a complete structural analysis.^5^ Historically speaking the preparation of HG 14 enolates was mainly triggered by the need of substrates for spectroscopic studies on these compounds. Quite recently the Stueger group successfully isolated the first tetraacyl substituted germanes and stannanes, and showed their ability to serve as long‐wavelength photoinitiators with superior potential.^6^, ^7^ During these reactions the key intermediates are HG 14 enolates, which allows a straightforward access to these highly desirable compounds. Another milestone in the chemistry of HG 14 enolates was the report of the first sila‐aldol reaction, which emphasizes the tight connection between silicon and carbon chemistry.^8^ This new synthetic strategy must be considered as a powerful alternative to standard coupling techniques, such as the Wurtz reaction,^9^ hydrosilylation,^10^ as well as transition‐metal‐catalyzed silicon‐carbon coupling reactions.^11^ Moreover, this novel synthetic method provides a straightforward access to structurally complex silicon frameworks, in quantitative yield. With these findings, HG 14 enolates demonstrate their importance and applicability in a broad range of chemical transformations.

**Scheme 1:**
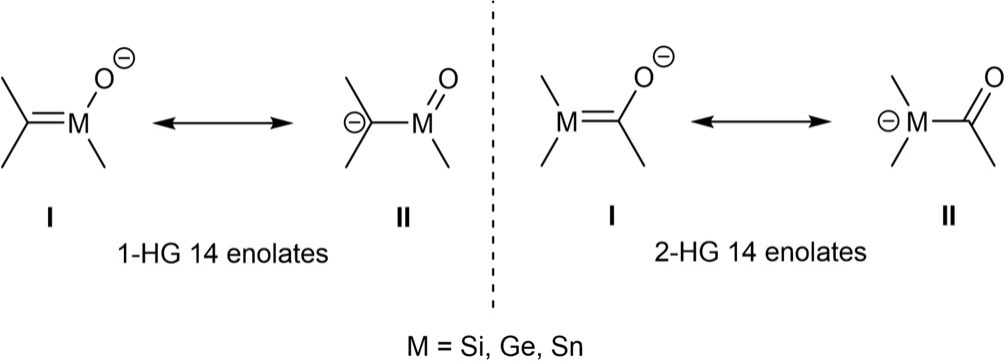
Resonance structures of HG 14 enolates.

HG 14 enolates can exist in two possible isomeric structures (Scheme 1). 1‐HG 14 enolates are still undiscovered, due to the low stability of a metal‐carbon double bond.^12^ With regard to 2‐HG 14 enolates, numerous reports on this compound class exist. As for metal enolates, two resonance structures for 2‐HG 14 enolates are possible: in the enol form (I), the negative charge is primarily located on the oxygen atom, whilst in the keto form (II) the negative charge resides predominantly on the silicon atom (Scheme 1).^13^, ^14^ The dominant structure of metal enolates is generally the enol form and preferably occurs in solid state, as well as in solution.^14^ HG 14 enolates show a significantly different resonance behavior. The position of the equilibrium is strongly influenced by the chosen alkali metal, the solvent system, as well as the substituent at the carbonyl‐moiety.

In this Minireview, we first present the most important strategies reported towards the synthesis of heavier Group 14 enolates with a particular emphasis on structural assignments and spectroscopic behavior. Then we focus on the recent advances in this field and give a brief outlook.

## 2 Silenolates

### 2.1 Lithium‐silenolates

**Scheme 2:**
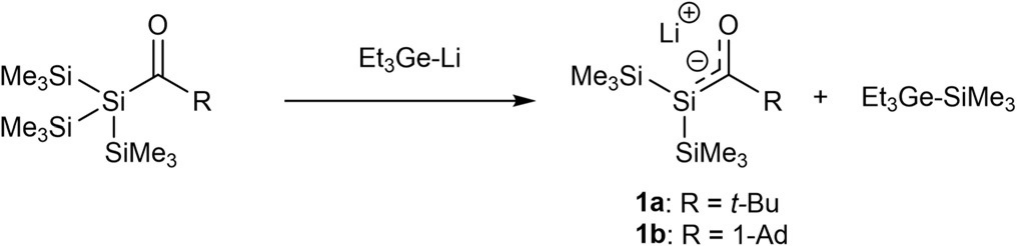
Synthesis of lithium‐silenolates by Bravo‐Zhivotovskii.

The first synthesized silenolates were lithium‐silenolates by the group of Bravo‐Zhivotovskii.^4^ They reported on the synthesis of silenolates and introduced the general strategy of reacting a germyl‐lithium reagent with an acylsilane in order to generate silenolates (Scheme 2). These silenolates were found to be relatively unstable, with a half‐life time of approximately 12 h. However, the decomposition products resulting from **1** were not identified.

**Scheme 3:**
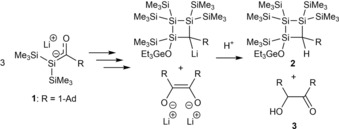
Degradation process of the lithium‐silenolate **1**.

In a follow up paper, Apeloig and Bravo‐Zhivotovskii succeeded in the identification of a possible degradation process of **1**.^15^ They found that an excess of the used base (e.g., two‐fold excess) leads to the formation of a 1:2 mixture of the trisilacyclobutane **2** and of (adamantoyl)adamantylcarbinol **3**. (Scheme 3. Note: Stirring for 48 h at room temperature followed by aqueous work‐up). The mechanism is rather complex and involves three silenolate moieties, as well as a Peterson elimination in order to obtain compound **2** and **3**. For the complete mechanism the reader is referred to the original publication.^15^

**Scheme 4:**
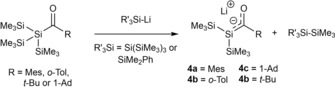
Synthesis of lithium‐silenolates by Ohshita and Ishikawa.

J. Ohshita and M. Ishikawa expanded this strategy and introduced more precursor molecules, as well as the use of different lithium reagents.^16^, ^17^ Additionally, they extensively explored the chemistry of their synthesized lithium‐silenolates. During the course of their studies concerning the chemical reactivity of acylpolysilanes with organolithium reagents, they found that the reaction of acylpolysilanes with silyllithium reagents resulted in the formation of lithium‐silenolates **4 a**–**d** in solution (Scheme 4). These lithium silenolates are thermally instable. **4 a** is moderately stable at room temperature. **4 b** undergoes a fast degradation even at temperature below −80 °C. **4 c**,**d** are more stable than **4 b**, but undergo uncharacterized degradation processes at room temperature. Therefore, all chemical manipulations were performed in situ.

**Scheme 5:**
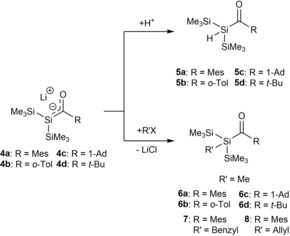
Reactivity of **4 a**–**d** with H_2_O and selected examples of alkyl‐, benzyl‐ and allyl‐halides.

The reaction of **4 a**–**d** with H_2_O resulted in the formation of bis(trimethylsilyl)acylsilanes **5 a**–**d** in nearly quantitative yields (Scheme 5). The reaction of **4 a**–**d** with alkyl halides led to the formation of alkylated acylsilanes in all cases. Additionally, **4 a** was also reacted with allyl and benzoyl halides. Again the lithium‐silenolate **4 a** reacts at the silicon center to give benzylmesitoylbis‐(trimethylsilyl)silane (**7**) and allylmesitoylbis(trimethylsilyl)silane (**8**) in good yields.

**Scheme 6:**
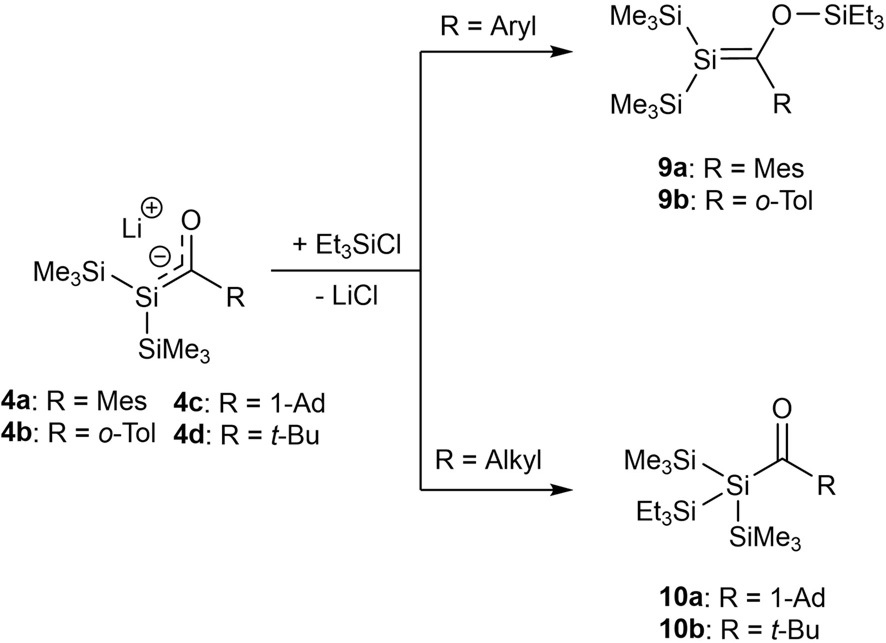
Reactivity of **4 a**–**d** with chlorosilanes.

Metal enolates are known to react with chlorosilanes under the formation of a silyl enol ether.^1^, ^18^ Ohshita and Ishikawa further studied the reactivity of **4 a**–**d** towards the reaction with chlorosilanes. Interestingly, the reactions of lithium‐silenolates **4 a**–**d** with chlorosilanes underwent two different pathways. The chosen pathway is dependent on the substituent at the carbonyl group. **4 a**,**b**, which bear an aryl substituent at the carbonyl group, form the Brook‐type silenes **9 a**,**b**. On the other hand **4 c**,**d** with alkyl groups at the carbonyl group form the acylsilanes **10 a**,**b** (Scheme 6). The cause for the different reactivity was determined by NMR spectroscopy. The negative charge in **4 c**,**d** is moderately localized on the central silicon atoms, whereas in **4 a**,**b** the negative charge is effectively delocalized over the silicon atoms and carbonyl groups. This reactivity was also found by other groups, which will be discussed in more detail later in this review.

**Scheme 7:**
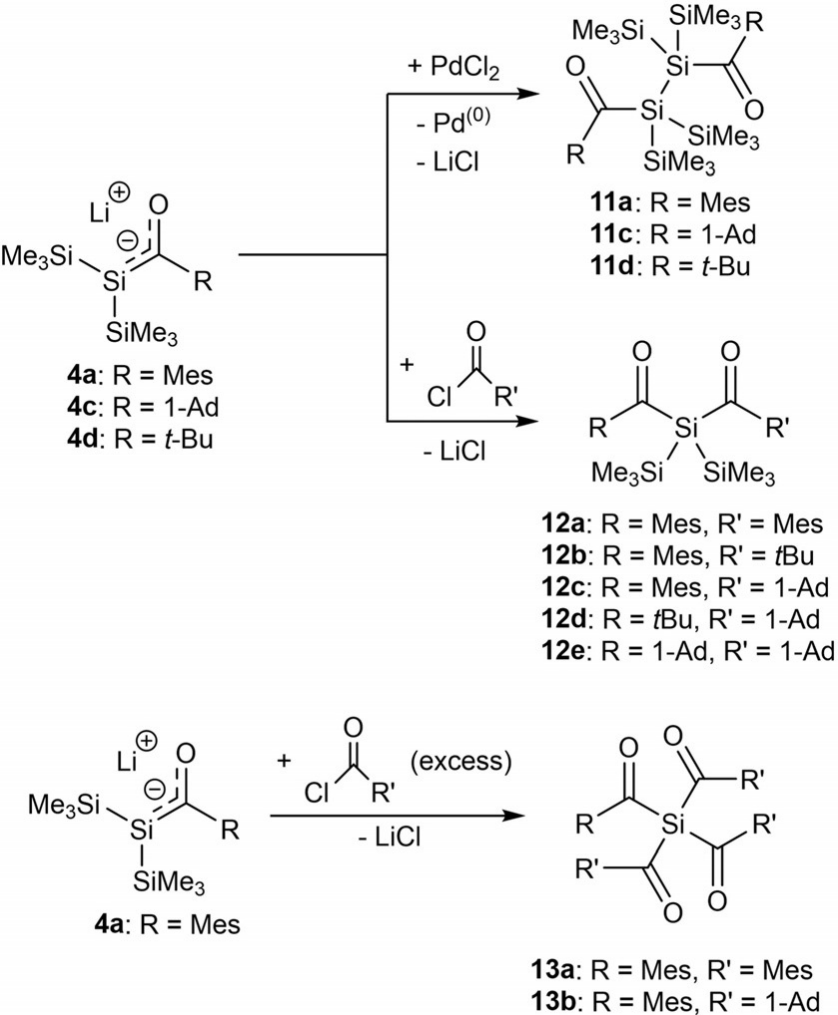
Reactivity of **4 a**,**c**,**d** with acid chlorides.

Furthermore, they also demonstrated that oxidative coupling of lithium‐silenolates with palladium(II) chloride leads to the formation of bis(acyl)polysilanes **11 a**,**c**,**d**.^19^ This was the first example of polysilanes with two silicon‐acyl bonds on the adjacent silicon atoms (Scheme 7). Moreover, they reacted their lithium‐silenolates with various acid chlorides and obtained the first examples of di‐ and tetraacylsilanes **12 a**–**e** and **13 a**,**b** (Scheme 7).^20^

**Scheme 8:**
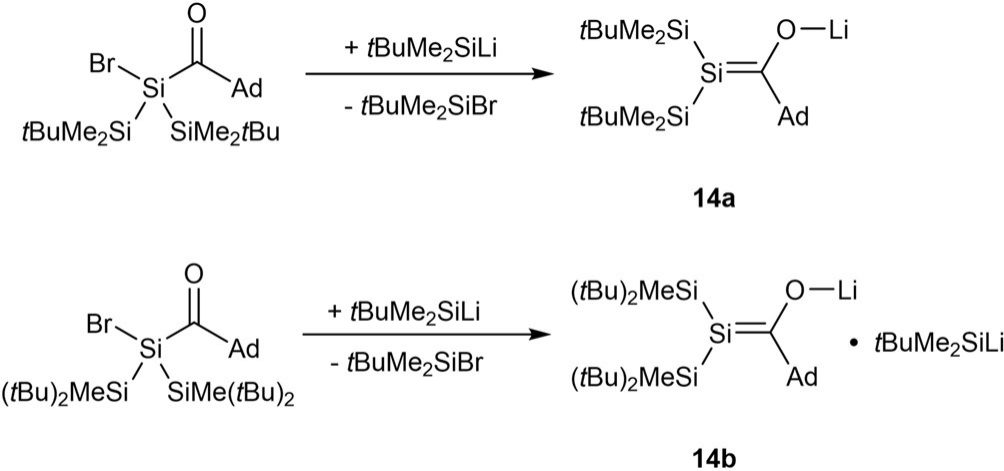
Synthesis of lithium‐silenolates.

**Figure 1:**
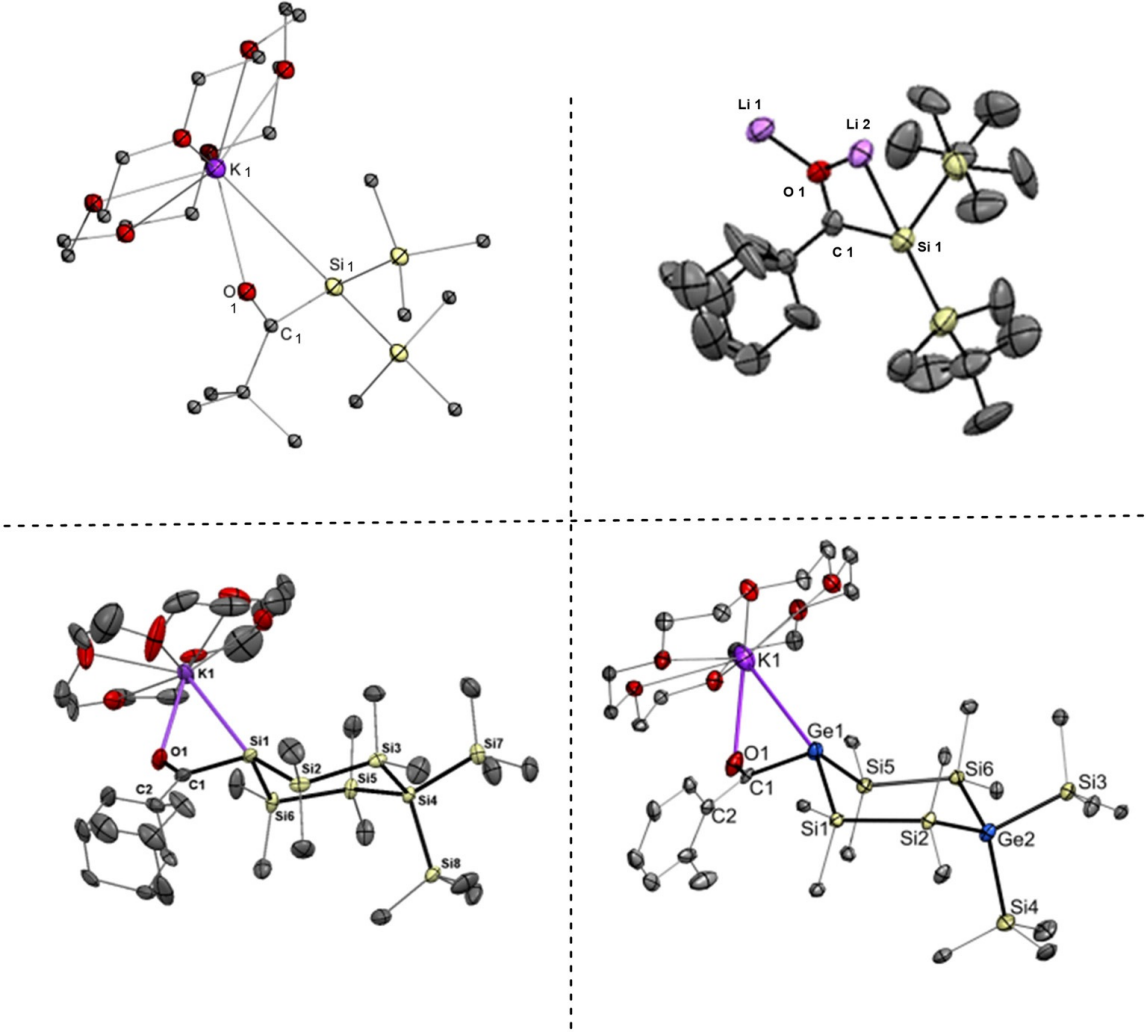
Selected examples of single‐crystal X‐ray structures of HG 14 metal enolates. (Top Left: potassium‐silenolate **15**; Top Right: lithium‐silenolate **14 a**; Bottom Left: cyclic potassium‐silenolate **18 c**; Bottom Right: cyclic potassium‐germenolate **25 b**).

Recently Apeloig and co‐workers used a different approach towards the generation of lithium‐silenolates. They synthesized them by metal‐halogen exchange between silyl‐lithium reagents (in excess) and bromo‐acylsilanes in hexane. With this methodology they were able to isolate their silenolates and to obtain X‐ray molecular structures of the first enol‐form silenolates **14 a**,**b** (Scheme 8).^21^ The X‐ray structure is depicted in Figure 1 and will be discussed Section 5.

In a follow up paper this group also investigated the reactivity of their isolated silenolates **14 a**,**b**. Upon addition of a polar solvent such as THF an interesting rearrangement occurred and lithium‐silenides were formed.^22^ For the complete mechanism the reader is referred to the original publication.

### 2.2 Potassium‐silenolates

**Scheme 9:**
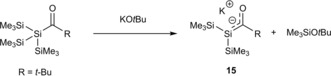
Synthesis of potassium‐silenolates.

A very important milestone in the chemistry of HG 14 enolates was the introduction of potassium as counterion by Ottosson and co‐workers. They reacted tris(trimethylsilyl)acylsilane with potassium *tert*‐butoxide (KO*t*Bu) and observed the quantitative formation of a potassium‐silenolate (Scheme 9).^5^ In contrast to the more difficult preparation of lithium‐silenolates, these silenolates are found to be thermodynamically more stable, and thus could be stored under an inert atmosphere and ambient temperature over a few months without degradation. This stability allowed Ottosson to obtain the first X‐ray structure of a silenolate reported in the literature. The molecular structure is depicted in Figure 1 and will be discussed in Section 5.

**Scheme 10:**
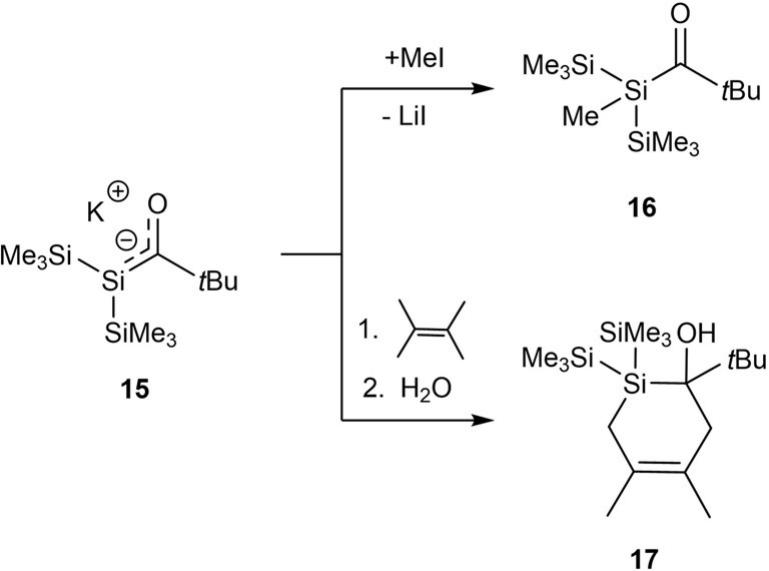
Reactivity of **15** with MeI and 2,3‐dimethyl‐1,3‐butadiene.

They also briefly investigated the reactivity of **15**. The trapping of **15** with MeI leads to the formation of the silicon‐methylated product **16**. The reaction of **15** with 2,3‐dimethyl‐1,3‐butadiene yields exclusively to the formation of the [4+2] adduct **17** (Scheme 10). Ohshita and Ishikawa found the same reactivity for their lithium‐silenolates.^16^

**Scheme 11:**
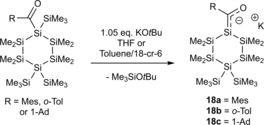
Synthesis of cyclic potassium‐silenolates.

Later on, the Stueger group demonstrated the possibility of synthesizing and characterizing cyclic silenolates **18 a**–**c**.^23^ **18 a**–**c** were obtained with remarkable selectivity by the addition of 1.05 equiv of KO*t*Bu to the corresponding acylcyclohexasilanes either in THF or in toluene solution in the presence of 1.05 equiv of [18]‐crown‐6 ([18]‐cr‐6) at −50 °C (Scheme 11). In the absence of air these cyclic potassium‐silenolates have the same stability as the acyclic derivatives. However, **18 a**–**c** decompose immediately to uncharacterized material upon exposure to the atmosphere, or the attempted removal of the solvent and other volatile components in vacuum. Nevertheless, they obtained crystal structures for **18 b** and **18 c**.

**Scheme 12:**
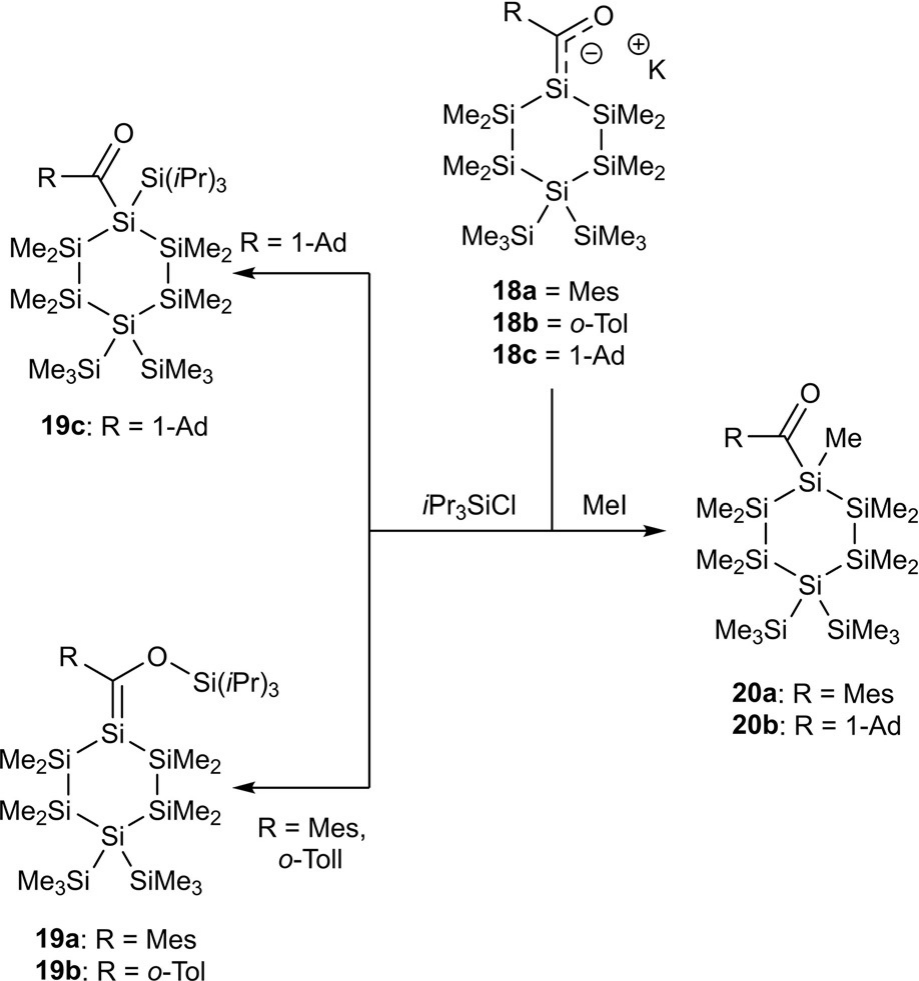
Reactivity of **18 a**‐**c** with chlorosilanes and MeI.

Furthermore, they studied the reactivity of **18 a**–**c** towards the reaction with chlorosilanes and MeI. They found the same reactivity as for acyclic lithium silenolates (see Scheme 12). The reaction of **18 a**,**b** with chlorosilanes allowed the formation of exocyclic silenes **19 a**,**b**. The reaction of **18 c** with chlorosilanes give rise to the formation of acylcyclohexasilane **19 c**. In the reaction of **18 a**,**c** with MeI, same reactivities in terms of reaction sites were found. In both cases, alkylation of the silicon atom was observed in nearly quantitative yields.

**Scheme 13:**
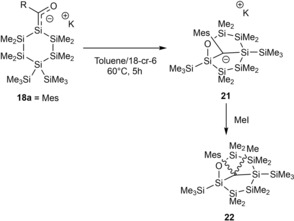
Rearrangement cascade to the substituted carbanion **21** and its subsequent trapping with MeI to **22**.

In a follow up paper they also examined the thermal stability of **18 a**,**c**.^24^ A selective rearrangement cascade was found when **18 a** was stirred for 5 h at 60 °C leading to the formation of highly interesting carbanion 2‐oxahexasilabicyclo[3.2.1]octan‐8‐ide **21**. This observation indicates that **18 a** was only the kinetic product, which thermodynamically rearranged through a mild, selective silyl‐migration cascade to the bicyclic carbanion **21** (Scheme 13). This cascade represents the first example of an intramolecular Sila‐Peterson reaction, where the formed silene is trapped by the present oxygen nucleophile intramolecularly (For the complete mechanism the reader is referred to the original publication).^24^ Upon the addition of MeI to a freshly prepared toluene solution of **21**, the corresponding methylated bicyclic adduct **22** was formed in the diastereomeric ratio of *endo*:*exo*=2:1.

Interestingly the same reaction set‐up for **18 c** led to complete degradation of **18 c** to uncharacterized material. They assumed that, in the case of alkyl‐substituted systems, the negative charge could not be distributed in the same way as in compound **21** and the primarily formed carbanion reacted further under the applied reaction conditions.

## 3 Germenolates

### 3.1 Lithium‐germenolates

**Scheme 14:**
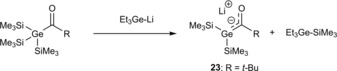
Synthesis of lithium‐germenolate.

Again the first synthesized germenolate was a lithium‐germenolate by the group of Bravo‐Zhivotovskii.^4^ They reported briefly on the formation of this lithium‐germenolate **23** from the reaction of an acylgermane with Et_3_GeLi (Scheme 14). Spectroscopic and structural data of **23** were not given.

### 3.2 Potassium‐germenolates

**Scheme 15:**
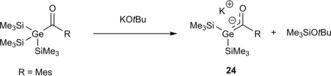
Synthesis of potassium‐germenolate **24**.

Potassium‐germenolates were found to be crucial intermediates for the synthesis of tetraacylgermanes.^6^ To verify this assumption tris(trimethylsilyl)acylgermane **24** was reacted with 1.05 equiv. of KO*t*Bu (see Scheme 15). A quantitative formation of the corresponding germenolate was observed. The molecular structure of **24**, as determined by single‐crystal X‐ray crystallography, and the complete set of consistent NMR data can be found in Section 5

**Scheme 16:**
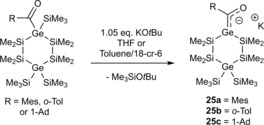
Synthesis of cyclic potassium‐germenolates.

Our group also succeeded in the isolation and characterization of the first cyclic germenolates. Therefore, the corresponding acyl‐1,4‐digermacyclohexasilanes were reacted with 1.05 equiv. of KO*t*Bu at −70 °C (see Scheme 16).^25^ The stability of these cyclic germenolates is comparable to their silicon homologs. After addition of [18]‐cr‐6 in toluene, we were able to grow crystals of the 1:1 [18]‐cr‐6 adducts of **25 b** and **25 c**, which were suitable for single‐crystal X‐ray crystallography (see Section 5).

**Scheme 17:**
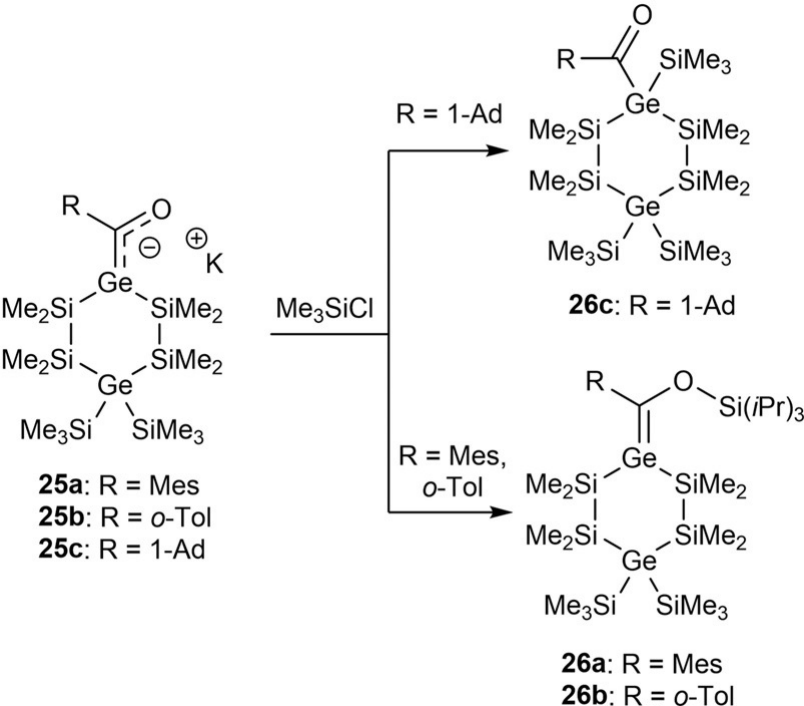
Reactivity of **25 a**–**c** with chlorosilanes.

The reactivity of **25 a**–**c** versus chlorosilanes parallels that observed earlier for silenolates. Thus, **25 c**, with an alkyl group attached to the carbonyl moiety, reacted with Me_3_SiCl at 0 °C under formation of the corresponding cyclic acylgermane **26 c**, while the aryl‐substituted compounds **25 a**,**b**, under the same conditions, exclusively afforded the O‐silylated germenes **26 a**,**b** (Scheme 17).

**Scheme 18:**
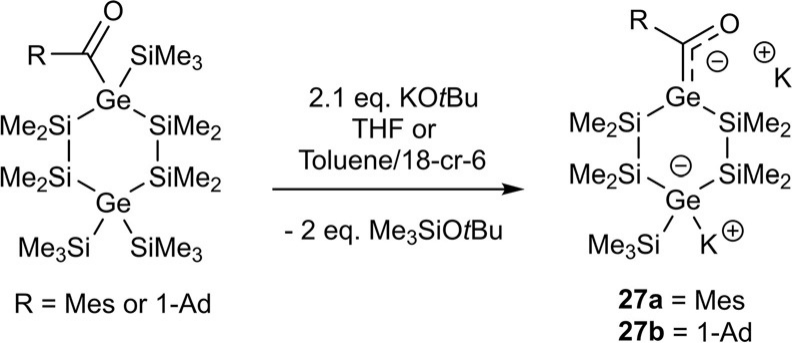
Synthesis of cyclic dianionic potassium‐germenolates.

Furthermore our group demonstrated the possibility to generate the first examples of dianionic germenolates **27 a**,**b**, which were synthesized by the reaction of the corresponding cyclic acylgermanes with 2.1 equiv. of KO*t*Bu (see Scheme 18). After addition of [18]‐cr‐6 in toluene we were able to grow crystals of the 1:2 [18]‐cr‐6 adducts of **27 a** and **27 b**, which were suitable for single‐crystal X‐ray crystallography (see Section 5).^26^

## 4 Stannenolates

No stable stannenolates are reported so far. Recently our group published a paper on previously unknown tetraacylstannanes. During their formation, stannenolates were found to be crucial intermediates.^7^

## 5 Characterization and Bonding in Group 14 Enolates

In the following section, the spectroscopic behavior, as well as important structural features of HG 14 enolates will be discussed. Moreover, a short summary of theoretical studies concerning HG 14 enolates will be given.

### 5.1 NMR spectroscopy

The chemical shift of the central metal atom for HG 14 enolates is strongly influenced by the dominant resonance structure, the solvent as well as the used counter ion. The ^29^Si NMR chemical shifts of the central silicon atom of silenolates, which adopt the keto resonance structure, were found in the region from *δ*=−59 to *δ*=−93 ppm. The measured ^29^Si NMR shifts are in the typical range for silyl anions.^27^ The use of crown ethers leads to an even stronger high field shift of the central silicon atoms (^29^Si chemical shifts of the Si atom of **15** are *δ*=−78.7 ppm in THF and *δ*=−93.8 ppm when [18]‐cr‐6 is present). The measured ^13^C NMR shifts of the carbonyl C atoms of HG 14 enolates adopting the keto resonance structure were found in the region of *δ*=262 to *δ*=274 ppm, which are typical for sp^2^ hybridization, and close to the ones measured for the corresponding acyl‐derivatives. The measured ^29^Si NMR shifts for the central silicon atom of the silenolate, which adopts the enol form is *δ*=8.4 ppm. This is significantly downfield shifted compared to keto derivatives, and in the region for ^29^Si shifts of silicon atoms of a Si=C double bond. No ^13^C NMR spectrum for this compound is reported. The exact values for the reported HG 14 enolates are depicted in Table 1.

**Table 1:** ^29^Si NMR shifts of the central silicon atoms and ^13^C NMR shifts of the carbonyl atoms.

Compd.	^29^Si NMR [ppm]	^13^C NMR [ppm]	Compd.	^29^Si NMR [ppm]	^13^C NMR [ppm]
**4 a**	−55.9^[a]^	262.7^[a]^	**18 c**	−70.0^[e]^	265.1^[e]^
**4 b**	−70.5^[a]^	274.1^[a]^	**24**	–	280.9^[e]^
**4 d**	−70.3^[a]^	274.3^[a]^	**25 a**	–	281.6^[f]^
**14 b**	8.4^[b]^	–	**25 b**	–	279.5^[f]^
**15**	−78.7^[c]^	271.1^[c]^	**25 c**	–	281.9^[f]^
	−93.8^[d]^	268.8^[d]^			
**18 a**	−73.1^[e]^	264.7^[e]^	**27 a**	–	282.45^[f]^
**18 b**	−73.1^[e]^	264.7^[e]^	**27 b**	–	282.78^[f]^

[a] measured in 70 % THF+30 % [D_8_]THF at −40 °C; [b] solid‐state isotropic NMR; [c] measured in [D_8_]THF at RT; [d] measured in [D_8_]THF with [18]‐cr‐6 at RT; [e] measured in C_6_D_6_ with [18]‐cr‐6 at RT; [f] measured in THF with D_2_O capillary at RT.

### 5.2 UV/Vis spectroscopy

To gain more insight into the electronic nature of silenolates, as well as of germenolates, our group recorded the absorption spectra of **18 a**,**c** and **25 a**–**c** and assigned the longest wavelength absorption through time‐dependent density functional in combination with the polarizable continuum model (TDDFT‐PCM) calculations at the B3LYP/6‐31+G(d,p) level. All HG 14 enolates exhibit an intense absorption maximum between 400 and 500 nm. According to calculations, these bands are unequivocally assigned to a HOMO→LUMO or a HOMO→LUMO+1 transition. The HOMOs mainly correspond to the p_z_ orbital of the metal atom with little variation in shape and energy. Upon excitation, electron density is displaced into the π*‐orbital of the carbonyl moiety (LUMO or LUMO+1). The LUMOs of the aryl‐substituted species showed additionally conjugation of the carbonyl and the aromatic π‐systems, which results in the observed bathochromic shifts of the corresponding absorption bands. The obtained experimental and computational data are summarized in Table 2. Therefore, silenolates **18 a**,**c** and **25 a**–**c** are best described as acyl metal anions (keto form in Scheme 1) in solution, irrespective of the nature of the R group attached to the carbonyl moiety. In contrast to that, Apeloig also calculated the HOMO orbitals for their silenolates with enol character and found that they have pronounced π‐character.^21^

**Table 2:** Experimental and TDDFT‐PCM B3LYP/6‐31+G(d,p)//B3LYP/6‐31+G(d,p) calculated absorption bands λ in THF for the potassium‐HG 14 enolates **18 a**,**c** and **24 a**–**c**.

	*λ*_max,exp_ [nm]	*λ*_max,calc_ [nm]	Assignment	
**18 a/Si**	448	429	p_z_→π* (CO/aryl)	HOMO→LUMO
**18 c/Si**	438	418	p_z_→π* (CO)	HOMO→LUMO
**25 a/Ge**	442	426	p_z_→π* (CO/aryl)	HOMO→LUMO
**25 b/Ge**	463	496	p_z_→π* (CO/aryl)	HOMO→LUMO
**25 c/Ge**	422	412	p_z_→π* (CO)	HOMO→LUMO+1

### 5.3 Structural assignments

All silenolates adopting the keto resonance structure, which were characterized by single‐crystal X‐ray crystallography, have potassium as the counter ion. Generally, they have a strong pyramidal central silicon atom with an elongated Si−C single bond. Two examples of these compounds are depicted in Figure 1. Selected bond lengths *d* [Å] and selected sum of valence angles ΣαSi_1_ and ΣαC_1_ [deg] for the silenolates are illustrated in Table 3.

**Table 3:** Selected bond lengths *d* [Å] and sum of valence angles Σα(Si1) and Σα(C1) [deg] for silenolates.

	**14 b**	**14 b**	**15**	**18 b**	**18 c**
*d* C_1_−Si_1_	1.822(7)	1.811(2)	1.926(3)	1.966(2)	1.874(2)
*d* C_1_−O_1_	1.315(7)	1.367(2)	1.245(3)	1.244(2)	1.260(2)
*d* M_1_−O_1_^[a]^	1.858(12)	1.858(2)	2.846(2)	2.743(1)	2.701(1)
*d* M_1_−Si_1_^[a]^	2.795(2)	2.871(4)	3.714(1)	3.603(2)	4.935(1)
Σα(Si_1_)	360.0	359.9	317.8	316.7	326.8
Σα(C_1_)	360.0	360.0	359.7	359.9	359.7

[a] **14 b**: M=Li; **15** and **18 b**,**c**: M=K.

All silenolates adopting the enol resonance structure, which were characterized by single‐crystal X‐ray crystallography, have lithium as counter ion. Generally, they have a planar central silicon atom and the central Si−C bond shows double bond character. One example is depicted in Figure 1.

All germenolates, which were characterized by single‐crystal X‐ray crystallography, have potassium as counter ion and adopt the keto resonance structure. Generally, they have a strong pyramidal central germanium atom with an elongated Ge‐C single bond. One example of these compounds is depicted in Figure 1. Selected bond lengths *d* [Å] and selected sum of valence angles ΣαGe1 and ΣαC1 [deg] for the germenolates are depicted in Table 4.

**Table 4:** Selected bond lengths *d* [Å] and sum of valence angles Σα(Ge1) and Σα(C1) [deg] for germenolates.

	**24**	**25 b**	**25 c**	**27 a**
*d* C_1_−Si_1_	2.004(7)	2.007(5)	2.063(2)	2.055(3)
*d* C_1_−O_1_	1.249(2)	1.236(6)	1.231(3)	1.252(4)
*d* K_1_−O_1_	2.782(10)	2.733(4)	2.740(2)	2.758(8)
*d* K_1_−Ge_1_	3.947(4)	3.855(6)	3.613(9)	3.423(2)
Σα(Ge_1_)	310.5	304.6	310.7	314.0
Σα(C_1_)	359.8	359.9	359.9	359.6

### 5.4 Theoretical studies

The keto–enol equilibrium in metal silenolates has also been investigated computationally. Apeloig et al. found that in non‐solvating media the enol‐form of the silenolate dominates. The effective solvation of the cation, for example, by crown ethers, results in the formation of the keto‐form.^21^ Additionally, Ottosson et al. revealed in a related study that coordination of a solvated metal ion to the oxygen atom in silenolates results in shorter Si−C bond lengths, a smaller degree of pyramidalization around the central silicon atom, and a lower charge difference at the carbon and at the silicon atom (Δ*q*(SiC)) compared to the naked silenolate.^28^

## 6 Recent Advances

The synthesis and characterization of HG 14 enolates was mainly triggered by fundamental investigations in the field of main group chemistry. This changed drastically when the Stueger group found an elegant synthetic protocol towards the synthesis of the first tetraacyl substituted germanes and stannanes. Moreover, these compounds showed their ability to serve as long‐wavelength photoinitiators with superior potential.^6^, ^7^, ^29^ During these reactions, the key intermediates are HG 14 enolates, which allows a straightforward access to these highly desirable compounds. Another important discovery in the chemistry of HG 14 enolates was the report of the first sila‐aldol reaction.^8^ In the following section of this review these recent advances will be summarized.

### 6.1 Tetraacylgermanes and ‐stannanes as photoinitiators

**Scheme 19:**
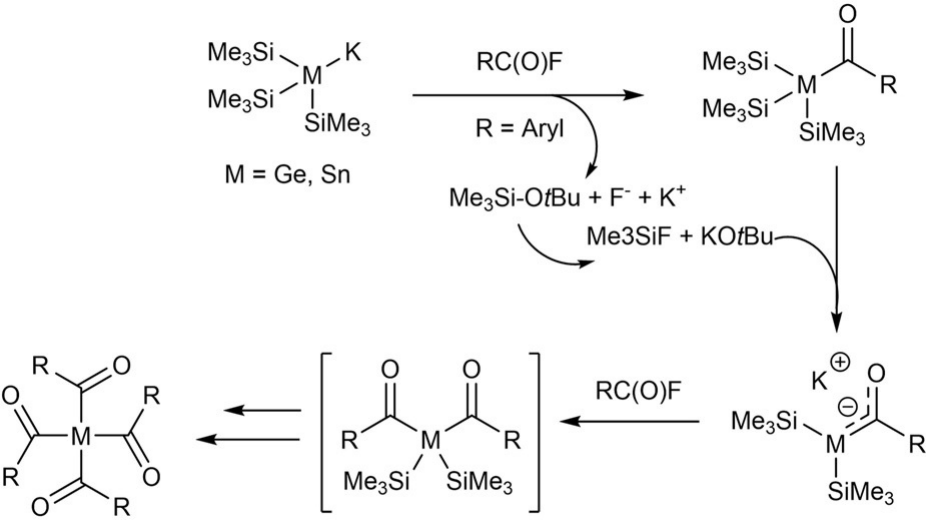
Mechanism for the synthesis of tetraacylgermanes and ‐stannanes.

Photoinitiators (PIs) play a very important role in a wide range of industrial processes (coatings, adhesives, dental filling materials, and the manufacture of 3D objects). Among the promising PI systems, tetraacylgermanes and tetraacylstannanes can act as suitable radical precursors generating acyl‐ and metal‐centered radicals upon irradiation, which add very rapidly to double bonds of various monomers.^30^ Moreover, they offer the advantages of significantly redshifted absorption bands and reduced toxicity compared to the frequently applied phosphorus‐based PIs.^31^ Furthermore, the synthetic protocol is very robust, one‐pot, and outperforms the methods in the synthesis of state‐of‐the‐art photoinitiators.^32^ The Stueger group discovered that the reaction of a potassium germanide and stannide with 4.1 equiv of acid fluorides leads to the exclusive formation of tetraacylgermanes or tetraacylstannanes in high yields. The mechanism is outlined in Scheme 19. HG 14 enolates were determined as crucial intermediates of this reaction.

### 6.2 Sila‐aldol chemistry

**Scheme 20:**
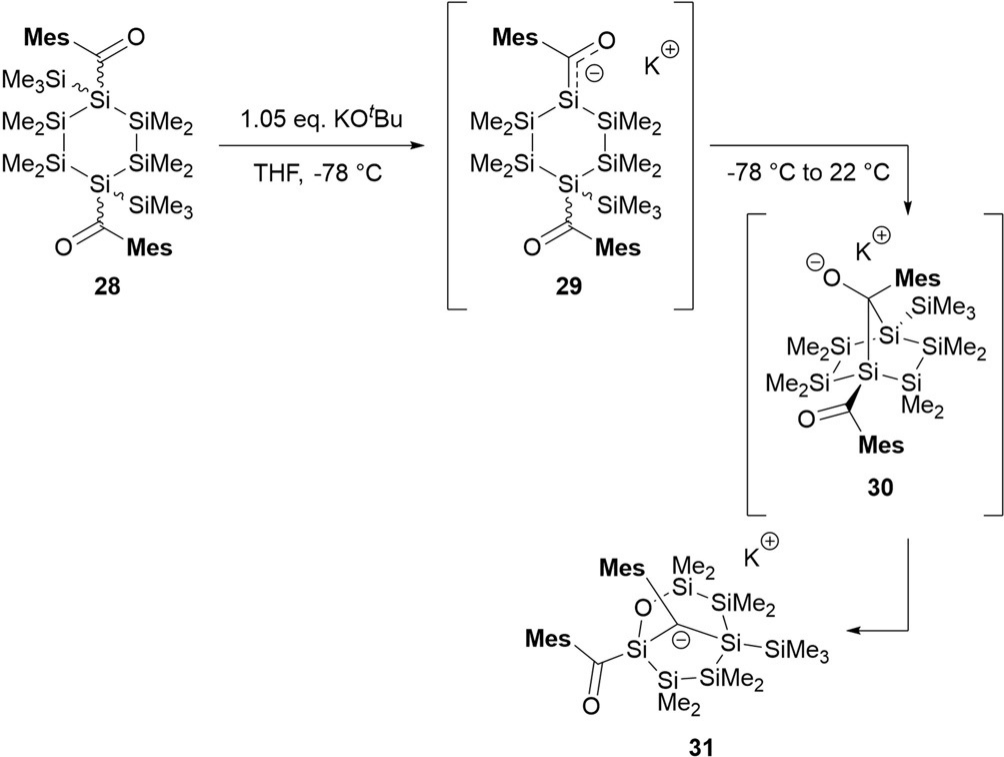
Sila‐aldol chemistry with the formation of complex silicon frameworks.

The classical aldol reaction, with its power in the reversible formation of carbon‐carbon bonds, is one of the most important organic reaction types.^2^, ^33^ The aldol reaction for silicon‐based compounds, were unknown until our group reported on the first sila‐aldol reaction. This reaction provides a straightforward access to structurally complex silicon frameworks. The starting point for the development of this transformation was the reaction of 1,4‐bis‐(acyl)cyclo‐hexasilane **28** with KO*t*Bu. As expected, the base abstracted one SiMe_3_ group to give monosilenolate **29. 29** immediately reacted further in the proposed sila‐aldol reaction. Interestingly, the silicon‐carbon addition product **30** was not directly observed by NMR spectroscopy, as this reaction selectively led to the formation of the bicyclic carbanion **31** in an ensuing anionic rearrangement cascade (Scheme 20). This transformation introduces a pioneering strategy for the formation of silicon‐carbon bonds by establishing a further link between the two related fields of silicon and carbon chemistry. The sila‐aldol reaction provides a significant addition to the synthetic methods available for the formation of a new class of silicon‐based compounds.

## 7 Conclusions and Outlook

Given that the chemistry of HG 14 enolates is relatively young, we have summarized in a concise and a complete way, the most important synthetic strategies towards this compound class. We have also shown their reactivity towards selected examples of electrophiles and trapping agents. Furthermore, we have summarized the spectroscopic behavior and the structural data for HG 14 enolates.

Inspired by the promising potential of tetraacylgermanes and ‐stannanes acting as long‐wavelength photoinitiators, we have highlighted their synthesis, where HG 14 enolates are crucial intermediates during their formation. More research towards the chemistry of this new photoinitiator class is likely to emerge soon.

The sila‐aldol chemistry has been shown to be highly efficient in the formation of complex silicon framework. This new synthetic strategy can be a powerful alternative to standard coupling techniques, such as the Wurtz reaction, hydrosilylation, as well as transition‐metal‐catalyzed silicon‐carbon coupling reactions.

## Conflict of interest

The authors declare no conflict of interest.

## Biographical Information

*Dr. Michael Haas graduated in chemistry at Graz University of Technology (Austria) in 2012 and received his Ph.D. in 2015 under the supervision of Prof. H. Stueger. From 2017–2018 he was working as a postdoctoral fellow at the Monash University in the group of Prof. C. Jones (Australia). Currently he started his independent career at Graz University of Technology (Austria). His research interests cover the synthesis of novel low‐valent Group 14 compounds and the design of new acyl Group 14 photoinitiators*.